# Moral encroachment and group-to-individual inferences

**DOI:** 10.1007/s11098-025-02412-x

**Published:** 2025-10-27

**Authors:** Martin Smith

**Affiliations:** University of Edinburgh, Edinburgh, United Kingdom

**Keywords:** Moral encroachment, Group-to-individual inferences, Justification, Probability claims

## Abstract

The paper is concerned with a special class of inferences, in which we draw conclusions about *individual people* based on evidence about the *groups* to which they belong. One thing that is notable about these inferences is that they are often subject to a kind of *moral* criticism. By judging people in this way, it is claimed, we demean or diminish them, and fail to properly respect them as individuals. And yet, if these inferences are epistemically sound – as they sometimes appear to be – then we face the possibility of a clash between moral and epistemic norms. One way to avoid this clash is through the thesis of *moral encroachment* – the idea that standards of epistemic justification are themselves sensitive to moral considerations and, in particular, can become more stringent when a belief has the potential to morally wrong others. In this paper I offer some reasons for doubting this thesis, and argue that group-to-individual inferences are, in the end, better understood through a more traditional framework on which epistemic justification is determined purely by the nature of one’s evidence.

## Moral/epistemic conflict

Since the early 2000s ‘love locks’ have become an increasingly common sight on prominent bridges, gates etc., affixed by couples who then dispose of the key as an expression of unbreakable love. Suppose that, while crossing a bridge one day, you pass a couple just as they attach a lock and drop the key into the rushing water below. You’ve never seen this couple before but, at a glance, you estimate that they would be no more than 17 or 18 years old, which prompts you to scoff, under your breath, at this gesture. You know that romantic relationships formed at this age seldom last. Perhaps you’re aware of research showing that 90% of adolescent romantic relationships eventually fall apart. As you walk away from the couple you form the belief that they will break up. What do we make of such a belief?

On the one hand your belief seems strongly supported by the evidence and is likely to be true. On the other hand, there seems to be something disrespectful – even patronising – about the way you blithely pass judgment on this couple. After all, you don’t really know anything about them *as individuals*. All you have is a statistic relating to young couples in general. By drawing this inference you are judging the couple based, in effect, on the behaviour of *others*, which seems unfair. If the couple were to learn of your belief, they would surely feel somewhat hurt and offended – and understandably so. A number of epistemologists have recently defended the view that we can wrong other people with our *beliefs* as well as our words and actions, and this particular belief would seem to be a strong candidate for such ‘doxastic wronging’^1^.

One might think that there is a collision here between moral and epistemic norms – your belief is morally criticisable because it wrongs the couple, but is epistemically justified because it is strongly supported by your evidence. The current example is similar to a number of prominent cases from recent epistemology, all of which involve *group-to-individual* inferences of some kind. Gendler (2011) discusses a case in which a diner in an elite social club believes that a person is an attendant on the grounds that he is African American, and the majority of African American people in the club are attendants and not members (see also Gardiner, 2018; Basu & Schroeder, 2019; Bolinger, 2020a, b). Moss (2018a, Sect. 10.4) discusses a case in which a consultant vising an office comes to believe that the person he passes in the hallway is an administrative assistant on the grounds that she is a woman, and the majority of the women who work in the office are administrative assistants (see also Gardiner, 2018, Johnson King & Babic, 2020).

Like the example of the couple, these two cases involve a move from statistical evidence about a group to a belief about an individual member of that group. These cases involve an additional factor in that the evidence in question may reflect patterns of systematic prejudice and discrimination. This, in turn, leads to an additional moral objection to the beliefs formed – that they serve to reinforce and perpetuate these very patterns (see Gendler, 2011; Moss, 2018b, Basu, 2019, Johnson King & Babic, 2020; Bolinger, 2020a, Sect. 4). In the example of the couple, though, these concerns are not I think at the forefront of our minds. The main objection we would make in this case – however difficult to precisely pin down – is that people are entitled to be treated as individuals, and not defined by their inclusion in a group.

One reaction to these cases is to conclude that moral and epistemic norms really *can* come into conflict – that it is possible to hold beliefs that are morally wrong but epistemically justified (Kelly & Roedder, 2008; Gendler, 2011). A different lesson we might draw from these cases is that we need to rethink the relationship between the moral and the epistemic. According to the thesis of *moral encroachment* the standards of epistemic justification or knowledge are sensitive to moral considerations and, in particular, can become more stringent when a belief has the potential to morally wrong others (Moss, 2018a, b; Basu & Schroeder, 2019; Bolinger, 2020a, Basu, 2021). Moral encroachment opens up the following diagnosis of the example of the couple: Although the belief that the couple will break up is justified ‘by ordinary standards’ – the standards that are operative when there are no moral considerations at play – it fails to meet the heightened standards that apply when moral wronging is in prospect. As a result the belief will be both morally wrong and epistemically unjustified^2^.

On closer inspection, though, it’s not clear that we need to resort to moral encroachment to avoid a moral/epistemic conflict in this example. There are various cases in which our evidence makes a proposition likely to be true but doesn’t seem to supply justification for believing it – and many of these cases lack any obvious moral dimension (Gardiner, 2018, Sect. 4). Suppose you’re about to roll a fair 10-sided die. Given your evidence, it’s 90% likely that the die will land on a number between 1 and 9 (inclusive). But would you be justified in believing that the die will land between 1 and 9? Many would take the view that you wouldn’t be justified in believing this – after all, the die could land on 10 just as easily as anything else and it seems you should remain open to this possibility.

One theory of justification that bears this out is the *normic* theory (Smith, 2010, 2016, 2018, 2022). Say that a body of evidence *normically supports* a proposition P just in case, given the evidence, the falsity of P would be abnormal, in the sense of requiring special explanation. If the vase on the table appears to you to be red then this provides normic support for the proposition that the vase *is* red. If the vase isn’t red, in spite of its appearance, then there would have to be some explanation for this – you’re undergoing a colour hallucination, there’s tricky red light illuminating the vase etc. If the sign in the shop window gives the opening time as 09.30 then that normically supports the proposition that the shop will open at 09.30. If 09.30 comes and goes and the shop remains shut then some explanation is needed – the sign is out of date and the shop has changed its hours, the owner is unwell or has been waylaid etc. In contrast, if you’re about to throw a fair 10-sided die then, while your evidence makes it likely that the die will land on a number between 1 and 9, it doesn’t provide normic support for this proposition. After all, the circumstance in which the die lands on 10 doesn’t require special explanation of any kind. According to the normic theory, one has justification for believing all and only those propositions that are normically supported by one’s evidence.

In the example of the couple, the evidence that 90% of young couples break up doesn’t normically support the proposition that this particular young couple will break up – they may simply be amongst the 10% that stays together, with no further explanation needed. There may be some temptation to think that if this relationship lasts when so many others falter, then there would have to be some special reason as to why – but, in truth, the evidence you possess doesn’t warrant any such assumption. Couples who reach significant milestones, such as 60th wedding anniversaries etc., are sometimes quizzed by journalists as to what their ‘secret’ is. But it’s instructive to note that they rarely report anything remarkable, or even anything that would really set them apart from countless other couples whose marriages fail. I would almost go as far as to say that, if a couple has expressed a commitment to one another (like the couple in the example) then what would require explanation is not the circumstance in which they stay together, but the circumstance in which they *break up*, no matter how common such occurrences may be^3^.

Another thing to note about the normic theory is that it predicts that justification is closed under deductive entailment – even when it involves multiple premises. Suppose propositions P and Q are each normically supported by your evidence and together entail a further proposition R. Since P is normically supported, the falsity of P would require special explanation. Since Q is normically supported, the falsity of Q would require special explanation. Since P and Q entail R, the falsity of R would require either the falsity of P or the falsity of Q in which case the falsity of R would also require special explanation and R would be normically supported (see Smith, 2016, Sect. 2.4, Chap. 4, 2018, 2022). This provides another way of showing that, according to the normic theory, you couldn’t possibly have justification for believing that the couple will break up. After all, if you did have justification for believing this, then you would have justification for believing the same thing about *any* arbitrary young couple (remember that, by stipulation, you don’t have any information that sets this young couple apart). If there are 100 locks on the bridge, all placed by young couples, then, given deductive closure, you would have justification for believing that *all 100* of these couples have either broken up or will break up – and that conclusion is clearly not justified by your statistical evidence.

If we adopt the normic theory, then we can avoid any clash between moral and epistemic requirements in this case, and can do so without appealing to moral encroachment. Your belief that the couple will break up is morally criticisable because it wrongs the couple and is epistemically criticisable because you don’t have the right kind of evidence to back it up. Similar remarks would apply to any case in which we form an outright belief about an individual person based purely on statistical evidence about a group to which they belong. The evidence that the majority of African American people in the club are attendants and the evidence that the majority of women who work in the office are administrative assistants will not normically support the conclusion that a particular person is an attendant or an administrative assistant^4^. These cases can all be handled within a more traditional framework on which epistemic justification is determined purely by the nature of one’s evidence. It’s just that we need to tease apart some of the different ways in which evidence can bear upon propositions^5^.

But this is not yet the end of the matter. Rather than believing that the couple *will* break up, what if you merely believed that the couple is *likely* to break up? Surely this belief would be justified on the basis of your evidence? Comparison with the dice example suggests so, as you clearly have justification for believing that the die is likely to land on a number between 1 and 9. Or consider another example involving a similar inference. Suppose a doctor judges that a patient is likely to have an adverse reaction to a particular medication, based on the fact that an adverse reaction was observed in 90% of those with a similar age, medical history, lifestyle etc. Surely the doctor would be justified in drawing this conclusion – indeed, it might seem negligent if they *didn’t* draw this conclusion^6^.

So if you limit yourself to the belief that the couple is likely to break up then, epistemically speaking, it seems that you would be hard to fault. Morally speaking, though, the situation remains murkier. While this belief doesn’t perhaps seem as morally problematic as a belief that the couple *will* break up, it doesn’t seem entirely unproblematic either. There is still a strong impression that you are reducing the couple to a particular age-bracket, and failing to properly treat them as individuals. The couple could still quite easily take offence at such a belief. So it looks as though a clash between moral and epistemic norms is once again in prospect – not over the outright belief that the couple will break up (where the moral and epistemic norms appear to agree) but over the probabilistic belief that the couple is likely to break up.

An advocate of moral encroachment can, seemingly, avoid this conflict in the same way as before. That is, they can say the following: Although the belief that the couple is likely to break up is justified by ordinary standards – the standards that apply in the dice example say – it falls short of the more demanding standards that take effect when moral wrongs are hanging in the balance. But unlike the original case, this now looks to be the *only* possible strategy for avoiding the conflict. The normic theory doesn’t appear to help – at least not straightforwardly. According to the normic theory you would have justification for believing that the couple is likely to break up just in case the falsity of this belief would require special explanation in light of your evidence. But what does it even *mean* for this belief to be false? It won’t be made false by the fact – if it is one – that the couple ends up staying together.

When it comes to the prospect of moral/epistemic conflicts we can distinguish between an ‘easy’ problem, which concerns cases of outright belief, such as the belief that the couple will break up, and a ‘hard’ problem, which concerns cases of probabilistic belief, such as the belief that the couple is likely to break up. The easy problem can be solved by looking closely at the nature of epistemic justification, and without departing from the conventional picture on which justification is purely a matter of evidence. But the hard problem looks as though it can only be solved by giving up the conventional picture and accepting moral encroachment. And discussions of moral encroachment often do revolve around the hard problem cases. Moss, for instance, discusses a version of the office case in which the consultant believes that the woman in the corridor is *likely* to be an administrative assistant (Moss, 2018a, Sect. 10.4, see also Gardiner, 2018).

Not only do the hard problem cases appear to offer a more persuasive argument for moral encroachment, they also broaden the potential *scope* of moral encroachment, pushing it further than even some moral encroachment *defenders* would be willing to go. Many of those who accept moral encroachment for outright beliefs may be inclined to deny that there is any moral encroachment on *credences* (for discussion see Pace, 2011, Fritz & Jackson, 2021, Gao, 2025, Enoch and Spectre, forthcoming, Sect. 2 ) which seems close to denying that there could be any moral encroachment on probabilistic beliefs, contrary to what the hard problem cases seem to require. I will return to the topic of credence in Sect. 3 below. In the end, I’m going to argue that even the hard problem can be solved from within the traditional view of justification and evidence^7^. But examining the conditions for epistemic justification won’t be enough^8^. Rather, it is to language – in particular *probabilistic* language – that we need to turn our attention.

## Probability claims

Suppose I’m balancing a glass bowl in a precarious way and you say ‘Be careful – that bowl will probably break if it’s dropped’. It’s natural to hear this as a claim about the *properties of the bowl* – what you’re saying is that the bowl is brittle or fragile or has a disposition to break if it strikes a hard surface. If, unbeknownst to you, the bowl is made of a specially hardened glass then your claim would be false. If you’re about to watch a tennis match and say ‘Both players are equally likely to win’ then it’s natural to interpret that as a claim about the ability of the competitors – like another way of saying that they’re evenly matched. If, unbeknownst to you, one of the players has an injury that will seriously hamper their game then, once again, the claim would be false.

But probability claims are not always interpreted like this. Suppose that, after carefully analysing the results of a medical trial, you report ‘This new drug is likely to be effective’. This would naturally be interpreted as meaning that there is strong *evidence* for the drug’s effectiveness. Even if, in actual fact, the drug is ineffective and the apparent effect observed in the trial was a result of random sampling error, this wouldn’t make your claim false. If someone said ‘The drug doesn’t work – why did you vouch for it?’ you would be within your rights to reply ‘I only said that the drug was *likely* to be effective – and that’s what the data suggested’. While your probability claim in this case could still turn out to be false – if, say, you were misinterpreting the data – it won’t be made false by the actual properties of the drug. The claim is not directly *about* the properties of the drug – rather, it’s about what your evidence *tells you* about the properties of the drug.

In some cases, a probability claim is used to say something about the dispositions or propensities of things in the world – call this the *objective interpretation*. In other cases, a probability claim is used to say something about the bearing or significance of a body of evidence – call this the *evidential interpretation*^9^. In many cases, both of these interpretations will be readily available, with neither one strongly preferred. Suppose you flip a coin and, as it spins through the air, you say ‘The coin is 50% likely to land heads’. On an objective interpretation, the claim expresses the proposition that the coin is disposed, when flipped, to come up heads as often as tails. If it turned out that the coin was actually weighted or double-headed, then the claim would be false. On the evidential interpretation, the claim expresses the proposition that your evidence provides equal support for the coin landing heads and for the coin landing tails. This claim would still be true even if, unbeknownst to you, the coin has some bias. If we think of a proposition as modelled by a set of possible worlds – i.e. the set of possible worlds in which it’s true – then the objective interpretation will correspond to the set of worlds in which the coin is fair, while the evidential interpretation will correspond to the set of worlds in which your evidence favours neither outcome over the other. While these sets partially overlap, they are largely distinct.

Another example: Suppose you know that half of the coins in a barrel are double-headed and half are fair. Suppose you choose a coin at random and flip it, without inspecting it. One thing you might say is ‘The coin is either 50% likely to land heads or 100% likely to land heads’. This cries out for an interpretation on which ‘The coin is 50% likely to land heads’ is true just in case the coin is fair and ‘The coin is 100% likely to land heads’ is true just in case the coin is double-headed. Another thing you might say is ‘It’s 75% likely that the coin will land heads’. That would not be naturally interpreted as a claim about the bias of the coin – no coins in the barrel are disposed to come up heads 75% of the time – but, rather, about the result of a simple probability calculation. If your evidence makes it 50% likely that the coin is fair and 50% likely that the coin is double-headed then it makes it 75% likely that the coin will land heads.

The interpretation of a probability claim can be influenced by considerations of charity, as in the preceding case, as well as a range of contextual factors. An objective interpretation would be preferred when questions about dispositions or characteristics are salient, as would often be the case when discussing *people*. An evidential interpretation would be preferred when discussing a body of evidence, and attempting to determine what it does and doesn’t show. There may also be cases in which the context offers no obvious steer, and the interpretation of a probability claim is left open and unsettled^10^.

Whatever the context, we can always force one or other interpretation by adding a suitable qualifying phrase. If you say ‘Given the nature of the coin, it is 50% likely to land heads’ then it’s clear that the intended reading is objective, and the truth of your claim hinges on whether the coin is fair, double-headed etc. If you say ‘Given my evidence, the coin is 50% likely to land heads’ then it’s clear that the intended reading is evidential, and any facts which go beyond your evidence – including facts about whether the coin is fair, double-headed etc. – are irrelevant to its truth^11^. By adding these qualifications we can even override our usual interpretive preferences. If you say ‘Given my evidence, that bowl will probably break if it’s dropped’ then you protect yourself from being refuted by any surprising discoveries about the bowl. In this case, your utterance will express the set of possible worlds in which your evidence strongly supports the proposition that the bowl will break if dropped. And this will include some worlds in which the bowl is, unbeknownst to you, made of hardened glass.

I won’t assume here that the objective and evidential interpretations are exhaustive, in the sense that these are the only available interpretations of a probability claim. In particular, I leave it open whether probability claims might also allow for a ‘non-propositional’ or ‘thoroughly probabilistic’ reading of the kind explored by Yalcin (2011) and Moss (2018a, partic. Chapters 2–3). But I will assume that the objective and evidential readings are amongst the available ways of understanding a probability claim^12^ and, more importantly for what is to come, can serve as the contents of beliefs. I will have more to say about the possibility of non-propositional readings in the next section.

By speaking of ‘objective’ and ‘evidential’ interpretations of probabilistic language, I may be inviting a comparison with the metaphysical project of ‘interpreting probability’ and, in particular, with the distinction between objective probability, often construed in terms of frequencies or propensities, and evidential probability, which is thought to measure the extent to which propositions are supported by a body of evidence (for an overview of this literature see Mellor, 2005). While this comparison may be helpful in some respects, I don’t think that the objective and evidential readings of a probability claim correlate with two different *kinds* of probability per se. Rather, the difference between the two readings lies in the set of facts – or *background conditions* – relative to which the probability is assessed^13^.

When one makes a claim like ‘Given the nature of the coin, it is 50% likely to land heads’ the probability will be assessed relative to the truth about the nature of the coin – at possible worlds in which the coin is fair, the probability will be assessed relative to the fact that the coin is fair, and at possible worlds in which the coin is double-headed, the probability will be assessed relative to the fact that the coin is double-headed and so on. The claim will be true at those worlds in which this probability works out to 50%. When one makes a claim like ‘Given my evidence, the coin is 50% likely to land heads’, the probability will be assessed relative to a different set of facts – the facts that comprise one’s evidence. The claim will be true at those possible worlds in which *this* probability works out to 50%^14^. While these claims have an overtly conditional form, the bare probability claim ‘The coin is 50% likely to land heads’ is still, in my view, used to express something about conditional probabilities – it’s just that the kinds of facts which enter into the background conditions will be determined by the context or, in some cases, left open and ambiguous.

While these remarks may point towards a general semantic framework for probability operators, I will leave the discussion informal here (and return to the issue in the appendix)^15^. Developing a formal semantics for probability operators is a significant project in its own right, but I don’t want to lose sight of the main goal – which is to resolve a certain kind of putative moral/epistemic conflict. In addition, I don’t want to give the impression that the arguments to come depend on any particular formal model – they don’t. The crucial point going forward is that when one is described as ‘believing that such-and-such is likely’, that description is ambiguous – it may indicate a belief about the propensities and dispositions that are out there in the world or it may indicate a belief about one’s own evidence.

## Resolving the conflict

The hard problem of moral/epistemic conflict centres on the belief that the couple is ‘likely’ to break up. But there are at least two different beliefs that could answer to this description. The first is an *objective* probabilistic belief – a belief that the couple is likely to break up, given their personalities, their history, the strength of their commitment etc. The second is an *evidential* probabilistic belief – a belief that the available evidence makes it likely that the couple will break up. While both beliefs might be expressed with the same words – i.e. ‘The couple is likely to break up’ – there are significant epistemic and moral differences between them. Once the two beliefs are carefully distinguished, the appearance of moral/epistemic conflict quickly fades – or so I will argue.

Suppose first that you hold an objective probabilistic belief. Would this belief be normically supported by your evidence – the evidence that 90% of young couples break up? In this case, the truth of your belief will hinge upon a series of facts about the couple. If the couple had not known each other long, and the decision to attach the lock was a relatively rash and impulsive one then, relative to these facts, it would be likely that the couple will break up. If, on the other hand, the couple know each other very well, have weathered crises, and are serious and sincere in their declaration then, relative to these facts, it would *not* be likely that the couple will break up. Whatever the case, though, your evidence doesn’t speak to these questions at all. To emphasise again: while you know a statistic about couples in general, you don’t know anything about *this particular couple*.

An objective probabilistic belief that the couple is likely to break up entails that the couple don’t have a sincere and abiding commitment to one another. And this assumption is not normically supported by any evidence that you possess. The fact that 90% of young couples break up doesn’t normically support any conclusion about the sincerity of the commitment of the two people that you pass on the bridge. The final thing to note is that the normic support relation, as discussed in Sect. 1, is closed under entailment. So if the objective probabilistic belief entails a proposition that lacks normic support, then the belief itself lacks normic support and, according to the normic theory, would be unjustified.

The present considerations can also help us to see why an objective probabilistic belief might raise *moral* concerns. If you form this belief about the couple then you are, in effect, judging their character and the authenticity of their devotion – and doing so without adequate evidence. The order of explanation here is the opposite from what the defender of moral encroachment would suggest – the fact that the belief is epistemically criticisable is part of the *reason* as to why it is morally criticisable (for related discussion see Gardiner, 2018, pp191-192, Begby, 2018, pp159-161). Similar remarks apply to the other examples of group-to-individual inferences. If the diner or the consultant forms an *objective* probabilistic belief that the person before them is likely to be an attendant/administrative assistant then they are unjustifiably judging the dispositions of someone they know nothing about, and doing so purely on the basis of their race or gender. The diner and consultant would be rightly morally criticised for taking such an attitude^16^.

What, then, if you form an *evidential* probabilistic belief as you pass by the couple? In this case, the *actual* facts about the history and personalities of the couple, and the sincerity of their commitment, will not enter into the background conditions – the set of facts on which the probability is conditionalised. Rather, this will be limited to the facts that constitute your current evidence and, by stipulation, these facts really *do* make it likely that the couple will break up. As a result, the evidential probabilistic belief is true and, indeed, should be *obviously* true, given the evidence that you possess.

Strictly speaking, the justificatory status of an evidential probabilistic belief will be determined by your *higher order* evidence – that is, your evidence *about* your evidence. While this can lead to complications in some cases, the present example would seem to be one in which your evidential situation is perfectly transparent. If the fact that the 90% statistic is part of your evidence is itself part of your (higher-order) evidence, and the fact that you don’t have further relevant evidence is itself part of your (higher-order) evidence, then the proposition that your evidence makes it likely that the couple will break up will itself be made *certain* by your (higher-order) evidence. So if you come to believe this then we don’t even get as far as asking whether its falsity would require special explanation in light of your evidence – its falsity is *ruled out* by your evidence. This belief would, as a limiting case, be normically supported and would count as justified according to the normic theory.

One might ask, at this point, where *credences* or subjective probabilities fit into this picture. How much credence should you invest in the proposition that the couple will break up? On one possible view, investing a credence in a proposition is just a matter of holding a corresponding evidential probabilistic belief. That is, to invest a high credence in a proposition is to believe that it is likely, given your evidence, that the proposition is true. To invest a precise credence – say 0.9 – in a proposition is to believe that there is a 0.9 probability, given your evidence, that the proposition is true. And so on. This is the familiar ‘belief-first’ approach to credence, on which credences reduce to beliefs about evidential probabilities (see for instance Lance, 1995, Weisberg, 2013, Moon, 2019, Moon & Jackson, 2020, Lennertz, 2024)^17^. If we accept this, then we get the result that you are justified in investing a high credence in the proposition that the couple will break up. While I am sympathetic to this view of credence, I don’t mean to presuppose it here, so it’s worth noting that we can get the same result from a weaker commitment: If one justifiably believes that a proposition has a certain probability, given one’s evidence, then one is justified in investing a corresponding credence in that proposition.

To sum up so far: There are two distinct probabilistic beliefs that you might express with the words ‘The couple is likely to break up’. The first is an *objective* probabilistic belief which would be morally wrong, but would also be epistemically unjustified. This is easily confused, however, with a second belief that really would be epistemically justified – namely, an *evidential* probabilistic belief. At this point, a defender of moral encroachment might insist that even an evidential probabilistic belief would wrong the couple, raising once again the spectre of moral/epistemic conflict. But this, I think, is something we should resist.

As discussed in Sect. 1, the main moral principle at work in this example is that people are entitled to be judged as *individuals* and not reduced to their membership in a particular group. But when it comes to an evidential probabilistic belief, there is a sense in which the belief is not really *about* the couple at all, at least not directly. Rather, it is a higher-order belief about your own evidence. Perhaps one way to see this is through the rough semantics of the last section; while an objective probabilistic belief determines a function from possible *couples* to truth values, an evidential probabilistic belief determines a function from possible *bodies of evidence* to truth values^18^. To make this more vivid we could imagine that you are explicitly suspending judgment on what the couple is like, on their history together etc. An evidential probabilistic belief that the couple is likely to break up can be perfectly well combined with a principled suspension of judgment on these questions^19^.

The couple could still take offence at your belief of course. They may be offended by the fact that you’re even *considering the question* of how likely it is, given your evidence, that they’ll break up – why not mind your own business? They may be offended by the fact that you even *have* evidence that makes their break up likely – where did you find statistics like this and why did you commit them to memory? But, given that you *do* have this evidence and you *are* considering the question, I don’t think the couple can have any further complaint about the conclusion that you reach – which is, after all, the *only* reasonable conclusion that you could have reached. While you might be morally criticised for gathering this evidence, or for considering this question, we shouldn’t confuse this with a moral criticism of the belief that you end up with (for related discussion see Enoch, 2016, pp24-26, Bolinger, 2020b, Sect. 1.2, Enoch & Spectre, 2021, Sect. 4.3, Ross, 2022, Sect. 5). Similarly, even if the diner or the consultant limits themselves to an evidential probabilistic belief, they might still be morally criticised for fixating on evidence that pertains to race or gender, and for considering the question of how likely it is, given this evidence, that the person in question is an attendant/administrative assistant. After all, there will be countless other questions about evidential probabilities that they *don’t* consider. But this is not the same thing as morally criticising the belief itself^20^.

Another thing to observe is that the claim under scrutiny here – namely, that the evidence makes it likely that the couple will break up – is a claim that I *myself* made when setting up this example in Sect. 1. And there’s nothing unusual in this – corresponding claims are often a crucial part of the commentary on examples of this kind (see for instance Gendler, 2011, p35, Basu & Schroeder, 2019, p200, Bolinger, 2020a, pp2515-2416). But there would be something very uncomfortable about us, as theorists, drawing attention to a key feature of an example while alleging that it would be morally wrong for the subject in the example to acknowledge *the very same feature*, even if it’s obvious to them.

Finally, even if we accept that an evidential probabilistic belief would be morally objectionable and are put under pressure to classify it as unjustified, it’s difficult to see how the machinery of moral encroachment could possibly deliver that result. Unlike an outright belief or an objective probabilistic belief, an evidential probabilistic belief doesn’t involve any inductive leap beyond one’s evidence – rather, it involves nothing more than careful reflection on the evidence itself. For the defenders of moral encroachment, moral considerations can oblige us to take certain doubts or error possibilities more seriously than we otherwise would – but, in the case of an evidential probabilistic belief, there *are* no doubts or error possibilities, as the belief doesn’t extend any further than the evidence itself.

To make this concern more precise, consider the following principle which Smithies terms *probabilistic accessibilism* (Smithies, 2019, p230):

If the evidential probability of P is n, then it is evidentially certain that the evidential probability of P is n.

For present purposes, we needn’t accept that this principle holds universally – it will be enough to grant that there are *some* cases in which evidential probabilities are ‘accessible’, and to grant that the present case could be among them. If so, then the evidential probabilistic belief will meet the highest epistemic standards, and there will be no scope for the standards to rise any higher, no matter what moral considerations might apply^21^. So even if we go along with the defenders of moral encroachment, and agree that moral factors can sometimes make a difference to the justificatory status of a belief, there is good reason to think that an evidential probabilistic belief, formed in the present case, would have to be an exception.

I have argued that there is nothing morally wrong with believing that your evidence makes it likely that the couple will break up. If we accept the view, mentioned above, on which credences reduce to evidential probabilistic beliefs, then we can derive the result that there is nothing morally wrong with assigning a high credence to the proposition that the couple will break up. As before, we can also derive this from a weaker principle: If it is not morally wrong for one to believe that a proposition has a certain probability, given one’s evidence, then it is not morally wrong for one to invest a corresponding credence in that proposition^22^.

Before concluding there is one final matter to consider. Sarah Moss has recently defended an influential view on which probability claims can express ‘thoroughly probabilistic contents’ which don’t reduce to propositions about evidence or to propositions about propensities and dispositions. On Moss’s view, a thoroughly probabilistic content is not a proposition *at all*, at least if we think of propositions as being sets of possible worlds – rather, it is modelled directly as a set of probability functions, each defined over sets of possible worlds. So a claim like ‘It's likely that the couple will break up’ may simply express the set of probability functions which assign a sufficiently high probability to the set of possible worlds in which the couple breaks up.

It is important to note, though, that the objective and evidential readings of a probability claim are still recoverable within Moss’s framework. Moss accepts that if we add an evidential restrictor to a probability claim and say something like ‘Given the evidence, it's likely that the couple will break up’ then that will express a straightforward proposition about the evidence, which would also be available as a content for belief. And Moss concedes that, even when the restrictor is missing, contextual factors could still push us towards an evidential interpretation (Moss, 2018a, Sect. 2.5). Moss doesn’t explicitly consider a locution like ‘Given their personalities and dispositions, it’s likely that the couple will break up’ but would I think be under pressure to say something similar; that this would express a standard possible-worlds proposition about the personalities and dispositions of the couple – and this proposition could also serve as the interpretation of a bare probability claim, if the context is right.

So even if we accept Moss’s view, the preceding remarks about the example would still apply: If you form an objective probabilistic belief then this could be morally criticised, whereas if you form an evidential probabilistic belief then this would be morally neutral. But what if the thing you believe is a thoroughly probabilistic content? If this belief were thought to be morally wrong then this could raise yet again the possibility of a moral/epistemic conflict. Moss claims that thoroughly probabilistic beliefs *can* morally wrong others (Moss, 2018a, Sect. 10.4, 2018b) and this forms a part of her case for moral encroachment. But I find the matter much more difficult to judge. I agree that it can seem morally problematic to *say* ‘It’s likely that the couple will break up’ or ‘It’s likely that the woman is an administrative assistant’, but this doesn’t really speak to the moral status of a thoroughly probabilistic belief. After all, we already have an independent explanation for this reaction – namely, that these utterances can be interpreted as expressing objective probabilistic beliefs. And it’s not just that these are *possible* interpretations – as noted in Sect. 2, questions about personalities and dispositions are often particularly salient when discussing *people*^23^.

In the example of the couple, one of the key questions for determining the moral status of the target belief was whether it entailed any propositions about the character of the couple. While an objective probabilistic belief does entail such propositions, an evidential probabilistic belief does not. This is part of the reason why the former belief, but not the latter, might be thought to infringe the couple’s right to be treated as individuals. But on this measure, a thoroughly probabilistic belief will fall on the same side as an *evidential* probabilistic belief. On Moss’s framework, no propositions about the couple’s character will be entailed by a thoroughly probabilistic content, and one could believe this content while remaining neutral on all such propositions^24^. Would we really morally criticise a person who held the thoroughly probabilistic belief that the couple is likely to break up, if that person also explicitly disavowed any judgments about their personality, the sincerity of their commitment etc.?

Moss shows how to embed the idea of thoroughly probabilistic content within a comprehensive and systematic framework – and I have only touched on a few aspects of this framework here. But my aim here is modest – to put a dampener on any attempt to use thoroughly probabilistic contents as a way of reviving the argument for moral encroachment. For this revival to succeed, one would need to first argue that holding a thoroughly probabilistic belief, in the cases under discussion, would be morally wrong. Further, in arguing this, one would have to limit themselves to considerations that are *specific* to thoroughly probabilistic beliefs, and which could not be explained through a conflation with objective probabilistic beliefs. While I can’t rule such an argument out, given that the very existence of thoroughly probabilistic contents is tendentious, I think it would be an especially difficult argument to make.

Some philosophers have appealed to cases of group-to-individual inferences in order to argue for moral encroachment – the claim that standards of epistemic justification are infused with moral considerations. In this paper, I have argued that these cases carry a different lesson. First, they illustrate the different ways in which a body of evidence might support the truth of a proposition. Second, they put a spotlight on the meaning of probability claims, and the different beliefs that they can be used to express.

## Appendix: a semantics for probability operators

A probability claim such as ‘It is likely that the couple will break up’ is analysed here as involving a sentential operator PROBABLY, which combines with the meaning of the embedded sentence ‘the couple will break up’ to give the meaning of the whole. My aim in this appendix is to outline a formal semantics for this operator which tallies with the rough remarks in the main text. The framework I develop can also be used to model more complicated probabilistic operators, such as those that involve probability comparisons and those that ascribe numerical probabilities – and I will mention some of these in passing. My approach is very similar to that outlined by Yalcin (2010, Sect. 5, see also Lassiter, 2010, Sect. 4.4) but, as I will discuss, there will be some differences in how the formalism is interpreted. There is also a connection with the contextualist semantics for epistemic modals proposed by Dorr and Hawthorne (2013).

Let propositions be modelled as subsets of a set of possible worlds W. To avoid unnecessary technicalities, assume that W is finite. Let Pr be a probability function defined over propositions. Formally, Pr is a function that takes the subsets of W to numbers in the unit interval and meets the following constraints: (i) Pr(W) = 1 (ii) For all X, Y ⊆ W if X and Y are disjoint then Pr(X ∪ Y) = Pr(X) + Pr(Y). Informally, Pr might be thought to represent the ‘intrinsic plausibility’ of propositions in something like the sense of Williamson (2000, Sect. 10.1). Conditional probability functions can be constructed from Pr in the usual way: For all X, Y ⊆ W, Pr_Y_(X) = Pr(X | Y) = Pr(X ∩ Y)/Pr(Y) (and is undefined in case Pr(Y) = 0)^25^.

I assume that context supplies a function ƒ from worlds to nonempty propositions (W to ℘(W)\{∅}) where ƒ(w) is the proposition on which the prior function Pr is conditionalised before entering into the truth conditions for the PROBABLY operator at w. In the main text I referred to this proposition as the ‘background conditions’. The background conditions play a similar role to a *modal base* in Kratzer’s semantics for probability operators and epistemic modals (Kratzer, 1977, 1991)^26^. Finally, I assume that context also supplies a threshold value t indicating the level at which the PROBABLY operator applies. The truth conditions for the PROBABLY operator can be given as follows:

$$w \in [\text{PROBABLY}\;\varphi ]{\text{ iff }}Pr _{{f\left( w \right)}} [\varphi ] > t $$

That is, PROBABLY ϕ will be true at w just in case the probability of ϕ, given the background conditions at w, is greater than t. Using these resources, it is easy to formulate truth conditions for the comparative probability operators that we find in constructions like ‘The two players are equally likely to win’ or the numerical probability operators that we find in constructions like ‘It is 50% likely that the coin will land heads’. We can write clauses like:

$$\begin{gathered} {\text{w}} \in [{\text{EQUALLY PROBABLE}}\;\varphi ,\psi ]{\text{ iff }}Pr _{{f\left( w \right)}} [\varphi ] = Pr _{{f\left( w \right)}} [\psi ] \hfill \\ {\text{w}} \in [{\text{5}}0\% {\text{ PROBABLE}} \;\varphi ]{\text{ iff }}Pr _{{f\left( w \right)}} [\varphi ] = 0.{\text{5}} \hfill \\ \end{gathered} $$

and so on. But I will focus on the PROBABLY operator here^27^.

As mentioned in the main text, the objective and evidential readings of a probability claim correspond to different choices of background conditions and show up, in the present framework, in different construals of the function ƒ. On an evidential reading ƒ represents a salient body of evidence which may, as a default, be the evidence possessed by the speaker. In this case ƒ(w) will be the set of worlds consistent with the evidence possessed by the speaker at w (alternately, the conjunction of the propositions included in the speaker’s evidence at w)^28^. It is along these lines that Yalcin interprets the function ƒ. In so doing, he follows a well established tradition in the semantics of epistemic modals, stemming again from the seminal work of Kratzer (1977, 1991). On an objective reading, however, ƒ may be better thought of as representing a *subject matter* – the nature of the coin, the character of the couple etc. Following Lewis’s well-known suggestion, a subject matter can, in turn, be modelled as a partition of the set of possible worlds W, in which any two worlds are grouped together just in case they agree with respect to the subject matter in question (see Lewis, 1988). In this case ƒ(w) will be the partition cell in which w is located – or, alternately, the set of worlds that agree with w regarding the subject matter in question^29^.

It’s natural to think that there are tight logical connections between probability operators and the epistemic modal operators ‘It must be that…’ and ‘It might be that…’. We can fit these operators into the present framework as follows:

$$\begin{gathered} {\text{w}} \in [{\text{MUST}}\;\varphi ]{\text{ iff}}\;\forall w^{\prime}\; \in f(w),\;w^{\prime}\; \in [\varphi ] \hfill \\ {\text{w}} \in [{\text{MIGHT}}\;\varphi ]{\text{ iff}}\;\exists w^{\prime}\; \in f(w),\;w^{\prime}\; \in [\varphi ] \hfill \\ \end{gathered} $$

This will give the result that PROBABLY is logically intermediate between MUST and MIGHT; [MUST ϕ] will be a subset of [PROBABLY ϕ] which will, in turn, be a subset of [MIGHT ϕ].

This suggests that epistemic modals might also be subject to objective and evidential readings. If ƒ is given an evidential gloss then this will align with the classic Kratzerian analysis of epistemic modals – in which they are seen as quantifying over the worlds that are compatible with a contextually determined body of evidence. If ƒ is given an objective gloss we arrive at something close to what Dorr and Hawthorne (2013) term ‘constrained’ readings of epistemic modals and Hawthorne (2007) terms ‘danger-theoretic’ readings.

## Footnotes

1 For the case in favour of doxastic wronging see for instance Basu (2018, 2019, 2021), Marušić and White (2018), Basu and Schroeder (2019). For criticisms see for instance Begby (2018), Enoch and Spectre (forthcoming). While I am sympathetic to the possibility of doxastic wronging, I won’t argue for it here. For the present problem to arise it is enough that beliefs can be subject to moral as well as epistemic evaluation, which is weaker than the claim that beliefs can constitute directed wrongs (see Bolinger, 2020b, Sect. 3.1, Enoch and Spectre, forthcoming, Sect. 1). But I will continue to speak of doxastic wronging in the main text.

2 Another class of cases which might be thought to involve a clash between moral and epistemic norms are those in which a person comes to hold a potentially hurtful belief about a close friend or loved one on the basis of what would usually be regarded as sound evidence (see, for instance, Stroud, 2006, Basu & Schroeder, 2019). These cases are also used to support the thesis of moral encroachment, but they don’t involve group-to-individual inferences (at least not obviously) and, thus, are beyond the scope of the present paper (for an insightful discussion of these cases see Gardiner, 2018, Silva, 2023, Sect. 7, Enoch and Spectre, forthcoming, Sect. 5).

3 If this thought is on the right track then the normic theory may even predict that you would be justified, in spite of the statistics, in believing *that the couple will stay together* on the basis of their commitment to one another – the very opposite of the cynical reaction in the original example. I won’t attempt to defend this stronger claim here, as it isn’t needed for present purposes. But if we did accept this, then the normic theory might have an application to the puzzle cases discussed by Marušić (2015) in which a person makes a promise or a resolution, in the face of evidence which shows that they are unlikely to follow through. Suppose, for instance, that you have resolved to quit smoking, but are aware of statistics showing that most attempts end in failure. Marušić claims – and I tend to agree – that you can’t rationally resolve to quit smoking unless you justifiably believe that you will succeed. But, given the discouraging statistics, how could such a belief ever be justified? Marušić’s suggestion, roughly speaking, is that the justificatory status of this belief is not determined by the quality of your evidence but, rather, by the quality of your practical reasons for making the resolution (Marušić, 2015, Chap. 6). But if the above reflections are right then the normic theory could offer an alternative way to secure the justificatory status of the belief – and one which doesn’t involve exempting it from general principles of epistemic evaluation.

4 For related discussion of the normic theory and group-to-individual inferences see Ma (2023, pp167-168) and Sridharan (forthcoming, Sect. 2 ). It’s important to note that we don’t have to commit to the normic theory in order to secure this result – there are alternative conditions on justification that could serve in its stead. Silva (2023, Sect. 7) argues for the same kind of conclusion on the grounds that, roughly, statistical evidence about a group can only justify a belief about an individual if it also justifies ascribing an associated disposition to that individual – a condition which is not met in the example of the couple, or in the diner and consultant examples. For what follows, I will continue to use the normic theory as my working account of justification, but I will have more to say about Silva’s view in n16.

5 If we change the evidence in these cases to something that *does* provide normic support, we would be much less inclined to morally criticise the resultant belief. Suppose you know the couple well, and one of them has confided in you that they’re planning on ending the relationship. In this case, it’s plausible that your evidence would provide normic support for the proposition that the couple will break up – but, in this case, there wouldn’t seem to be anything morally problematic about you believing this. Similarly, if we imagine that the diner or the consultant was *told* that the person in question was an attendant or an administrative assistant, and had no reason to doubt it, then we wouldn’t morally criticise them for taking that testimony at face value. For related discussion see Gardiner (2018, Sect. 4).

6 This kind of example is discussed by Pundik (2008, pp306-307), Buchak (2014, pp305-306) and Enoch and Spectre (2021, pp5690-5691).

7 This paper follows in the footsteps of Gardiner (2018) and Enoch and Spectre (forthcoming) who also argue that moral/epistemic conflicts can be avoided without needing to invoke moral encroachment. When it comes to the ‘easy’ outright belief cases, Gardiner and Enoch and Spectre pursue a similar strategy to the one I outlined above – that is, they emphasise the epistemic shortcomings of purely statistical evidence vis à vis outright beliefs (see also Silva, 2023, Sect. 7, Sridharan, forthcoming, Sect. 2 ). But when it comes to the ‘hard’ cases concerning beliefs about what is likely – which will be the focus of the remainder of the paper – our strategies are quite different. I’ll return to this in n8.

8 According to Enoch and Spectre, in order to form a justified belief about what is likely true of an individual, based on statistical evidence about a group, one also needs positive evidence to the effect that the individual is a *typical* member of that group (Enoch and Spectre, forthcoming, Sect. 4, see also 2021, Sect. 4.2). On this view, you couldn’t justifiably believe that the couple is likely to break up unless you had positive evidence that they were ‘a typical young couple’. But even if we had a solid grasp of what it means to be ‘typical’ member of a group, Enoch and Spectre don’t explain why beliefs of this kind should be subject to this extra evidential requirement. One immediate challenge comes from examples like the doctor case above. Surely the doctor, armed with the relevant medical statistics, doesn’t need to seek out positive evidence showing that the patient is a typical member of their cohort. To justifiably believe that the patient is likely to suffer an adverse reaction, it’s enough that there be no evidence of relevant *a*typicality. In any case, it’s difficult to speculate further about the evidential requirements for forming justified probabilistic beliefs without first getting clear on the *content* of such beliefs. It is to this that I turn next.

9 For a similar distinction with respect to epistemic modals see Hawthorne (2007), Dorr and Hawthorne (2013) and with respect to risk attributions see Smith (2023, Sect. 2).

10 In some contexts, the interpretation of a probability claim may even be underdetermined by the speaker’s *own* intentions. In these cases, I’m inclined to think that the effect of uttering a probability claim is to put both an objective and an evidential interpretation ‘into play’ in the sense that the claim could be retrospectively interpreted in either way, depending on the path the conversation takes. I won’t explore this here – but see von Fintel and Gillies (2011) for further related discussion.

11 We can use other evidential restrictors as well, saying things like ‘Given *our* evidence, the coin is 50% likely to land heads’, indicating that the probability is to be assessed relative to the combined evidence of all parties to the conversation. And we can even specify bodies of evidence that may be quite different from our own, as in ‘Given Keith’s evidence, the coin is 50% likely to land heads’. In a way, there are many possible ‘evidential’ readings of a bare probability claim like ‘The coin is 50% likely to land heads’ corresponding to different possible bodies of evidence. I’m inclined to think that the default evidential reading is the one that adverts to the evidence currently possessed by the speaker – but contextual factors can steer us elsewhere, even towards ‘exocentric’ readings that involve very different bodies of evidence (see for instance the cases in Egan, Hawthorne and Weatherson, 2005, Sect. 2 , Dorr & Hawthorne, 2013, Sect. 3.2 and Moss, 2018a, Sect. 2.5 ). In any case, when I speak of the evidential readings of probability claims, I have in mind the default ‘egocentric’ evidential readings, unless otherwise specified.

12 I’m inclined to think that, once we recognise the distinction between the objective and evidential readings, the case of favour of the non-propositional reading is significantly weakened, as the distinction can provide an alternative explanation for much of the relevant data. This topic is well beyond the scope of the present paper – but for some related discussion see Hawthorne (2007) and Dorr and Hawthorne (2013).

13 The third interpretation of probability that is usually placed alongside the objective and evidential interpretations is the *subjective* interpretation, on which probabilities measure one’s personal credence or confidence in a proposition. It may be that the subjective interpretation is also correlated with a possible reading of probability claims – on which they express propositions about the subjective probabilities that are assigned by the speaker. This would be a reading on which ‘The drug is likely to be effective’ would be true just in case you assign a high subjective probability to the effectiveness of the drug and, thus, could be true even if you were completely misinterpreting the relevant data. While I am doubtful that there is such a reading, I won’t take up this topic here, but I will have more to say about subjective probability in Sect. 3.

14 One might argue that, even on an objective interpretation, facts about the speaker’s evidence would still typically be included in the background conditions – it’s just that these conditions would also include extra-evidential facts about the dispositions and propensities of the subject of the claim. Here’s one reason for thinking this: Suppose the coin has landed and we can both clearly see that it’s showing tails. If, at this point, you were to say ‘It’s 50% likely that the coin landed heads’ then there doesn’t seem to be any felicitous interpretation of your claim, except perhaps an exocentric evidential reading (if, say, you are toying with someone who hasn’t seen how the coin landed). But the felicity of the claim cannot, I think, be rescued by suggesting an objective interpretation. Even the claim ‘Given the nature of the coin, it is 50% likely that it landed heads’ wouldn’t sound felicitous once you have seen the coin land tails. One plausible explanation for this is that the fact that the coin landed tails is now part of your evidence and, thus, a part of the background conditions, even on an objective interpretation (for similar suggestions regarding epistemic modals and risk attributions see, respectively, Dorr & Hawthorne, 2013 and Smith, 2023). This issue has no bearing on the discussion to come and I won’t consider it further here, though I will return to it briefly in the appendix.

15 The remarks made here fit with the general semantic approach to probability operators developed by Kratzer (1991) and others (for discussion see Yalcin, 2010). This approach is itself an extension of the standard contextualist semantics for epistemic modals – claims of the form ‘It might be the case that…’ and ‘It must be the case that…’. Further details are given in the appendix.

16 Silva (2023, Sect. 7) also observes that the diner’s evidence would not justify ascribing, to the person before them, any *disposition* to be an attendant. As discussed in n4 Silva regards this as a necessary condition for justifiably believing that the person is an attendant and, thus, concludes that the diner would not be justified in forming this belief. Silva doesn’t consider the case in which the diner forms the belief that the person is *likely* to be an attendant but, if the present reflections are right, then the same observation implies that this belief, if objective in nature, would also be unjustified.

17 On this view a subjective probability is, in effect, a subjective impression of an evidential probability, and subjective probability shouldn’t be considered a ‘kind’ of probability – any more than, say, a subjective impression of temperature should be considered a ‘kind’ of temperature. This view may also put some pressure on the idea, mentioned in n13, that probability claims could have a purely subjective reading, corresponding to the subjective interpretation of probability. I won’t pursue this further here.

18 If the belief you form is evidential then, in a way, it would be wrong to even characterise this as a case of ‘group-to-individual’ inference. While the surface form suggests a colloquial sense in which the premise is ‘about’ the group of young couples and the conclusion is ‘about’ the couple you are walking past, this doesn’t survive close scrutiny, which reveals that the reasoning remains one step removed from any individuals or groups; we have a premise about your evidence about a group and a conclusion about your evidence about individuals.

19 An evidential probabilistic belief can even be combined with an acknowledgement that one’s evidence is quite *limited* – that there is a lot of further relevant evidence that one doesn’t possess. We shouldn’t confuse the judgment that a proposition is likely, given one’s current evidence, with the judgment that one’s current evidence is in any way comprehensive or complete (for some related discussion see Smith, 2021, section V).

20 Having said that, depending on how we fill in the details of these examples it may be that the diner and consultant *are* making an epistemic error, in which case their beliefs could be morally criticised without inviting any conflict between moral and epistemic norms (for related discussion see Gardiner, 2018, Sect. 6). When it comes to the social club example in particular, it’s natural to assume that the diner *would* have further relevant evidence – such as the fact that the person in question is not wearing an attendant’s uniform. In this case, the proposition that the person is an attendant would not be likely, given the total evidence available to the diner, and any evidential probabilistic belief that they form to the contrary would be epistemically (and morally?) criticisable. The social club example is taken from the autobiography of the prominent American historian John Hope Franklin who writes ‘It was during our stroll through the club that a white woman called me out, presented me with her coat check, and ordered me to bring her coat. I patiently told her that if she would present her coat check to a uniformed attendant, “and all of the club attendants were in uniform”, perhaps she could get her coat’ (Franklin, 2005, p340).

21 A defender of moral encroachment might hold that moral considerations are capable, in some cases, of pushing the standards for justification *beyond the maximum*, so that they become impossible to meet. I think this idea is problematic, and resorting to it in the present case may bring particular difficulties – but I won’t explore this here (for some discussion see Hirvelä, 2024, Sect. 3 , Enoch and Spectre, forthcoming, p3, n10).

22 It is important to emphasise again that I am speaking of credence in the sense of *subjective probability*. When I say that there is nothing morally wrong with assigning a high credence to the proposition that the couple will break up, I mean that there is nothing morally wrong with assigning a high subjective probability – one that matches the believed evidential probability of the proposition. The term ‘credence’ can also sometimes be used to indicate the degree or the strength with which an *outright belief* is held (for some related discussion see Williamson, 2000, Sect. 4.4, Smith, 2016, Sect. 8.4). In this sense, one cannot assign a credence to the proposition that the couple will break up without believing it outright and, by the arguments of Sect. 1, this belief would be both morally and epistemically criticisable.

23 Unless there are contextual factors that strongly suggest an evidential interpretation, such utterances would, at the very least, serve to put an objective interpretation ‘into play’ in a way that could be picked up in the subsequent conversation (see n10). And even this, I would argue, is morally questionable.

24 Thoroughly probabilistic contents stand in set-theoretic relations which can subserve logical relations in the way that is already familiar from the possible-worlds model of propositions. So if one content is a subset of another then the former can be said to logically entail the latter, if two contents are disjoint then they can be regarded as logically inconsistent etc. Furthermore, a possible-worlds proposition P can also be represented in this framework – namely, as the set of probability functions such that P is true in every world in their domain (see Moss, 2018a, Sect. 1.4, 3.5, A.3). In this way, propositions can also enter into logical relations with thoroughly probabilistic contents. To see that the thoroughly probabilistic content that the couple is likely to break up will not entail any proposition about the personality or sincerity of the couple it suffices to point out that, for any such proposition, the probabilistic content will include functions that assign a non-extremal probability value to the set of worlds in which it is true. There are some wrinkles here however. In particular, even if a thoroughly probabilistic content fails to entail a proposition P it may still be the case that ∼P – where this corresponds not to the set-theoretic complement of P but to the set of probability functions in which P is false in every world in the domain – may still be inconsistent with that content (see Moss, 2018a, Sect. 4.4, A.5). So even if the thoroughly probabilistic content that the couple is likely to break up does not entail any propositions about the personality and sincerity of the couple it may nevertheless be inconsistent with some such propositions. But it remains the case that one could believe this content while remaining completely neutral on the propositions in question. I can’t pursue this further here.

25 Alternately, we could use a two-place conditional probability function, conforming to a suitable system of axioms (see for instance Rényi, 1955, Popper, 1959, appendix, Roeper and Leblanc, 1999, particularly Chap. 1, Sect.2 ). This would, in a way, fit better with the ideology of the present approach, on which probability claims invariably express conditional probabilities, even if the conditions are not made explicit. Nevertheless, the question of whether unconditional or conditional probabilities should be treated as primitive, for the purposes of axiomitisation, is a separate one – and not one that I consider here.

26 On Kratzer’s theory, probability operators are interpreted relative to two contextually supplied parameters – a modal base and an ordering source. Formally speaking, the modal base, at a world w, will be a set of worlds that includes w, while the ordering source will induce a transitive, reflexive relation on this set of worlds. This ordering of worlds will, in turn, induce an ordering of propositions, which is used to supply truth conditions for comparative probability operators at w (Kratzer, 1991, particularly Sect. 4). On one way of looking at things, the present approach deploys a probability assignment over the worlds in the modal base as a replacement for this ordering. For discussion of Kratzer’s theory see Yalcin (2010, Sect. 3).

27 If we want to allow for imprecise or interval-valued probabilities, we could begin with a *set* of probability functions, rather than a single function, each of which can be simultaneously conditionalised on ƒ(w). This will, amongst other things, allow for propositions to be incomparable with respect to their probability. I won’t give further details here.

28 If evidential consistency were required to be an equivalence relation then this would validate the *probabilistic accessibilism* principle from Sect. 3. In this case, for any w ∈ W and X ⊆ W if Pr_ƒ(w)_X = n then, ∀x ∈ ƒ(w), Pr_ƒ(x)_X = n. This condition can of course be met for a particular proposition at a particular world without our needing to impose this general constraint.

29 If we take up the suggestion floated in n14, on which the speaker’s evidence remains part of the background conditions, even on an objective interpretation, then we will need to reconfigure function ƒ in such a way that its output, at a world w, is the intersection of the outputs of the two functions mentioned in the main text. That is, ƒ(w) will represent the set of worlds which are consistent with the speaker’s evidence at w *and* which agree with w with respect to the subject matter in question.
